# Multifaceted Cardioprotective Potential of Reduced Glutathione Against Doxorubicin-Induced Cardiotoxicity via Modulating Inflammation–Oxidative Stress Axis

**DOI:** 10.3390/ijms26073201

**Published:** 2025-03-30

**Authors:** Amr Negm, Ezat A. Mersal, Amal F. Dawood, Amira O. Abd El-Azim, Omar Hasan, Rayan Alaqidi, Ahmed Alotaibi, Mohammed Alshahrani, Abdullah Alheraiz, Tamer M. Shawky

**Affiliations:** 1 Department of Chemistry, College of Science, King Faisal University, Al-Ahsa 31982, Saudi Arabia; 2 Department of Basic Medical Sciences, Vision Colleges, Riyadh 13226, Saudi Arabia; emersal@vision.edu.sa (E.A.M.); omartamim99@hotmail.com (O.H.); rayanalaqidi@gmail.com (R.A.); ahmedalotaibi217@gmail.com (A.A.); m.ayedh.sh@gmail.com (M.A.); abdullahalheraiz@gmail.com (A.A.); 3 Department of Basic Medical Sciences, College of Medicine, Princess Nourah bint Abdulrahman University, P.O. Box 84428, Riyadh 11671, Saudi Arabia; afdawood@pnu.edu.sa; 4 Zoology Department, Faculty of Science, Mansoura University, Mansoura 35516, Egypt; amirahosman2000@yahoo.com; 5 Department of Anatomy and Embryology, Faculty of Medicine, Cairo University, Giza 12613, Egypt; h.t.anatomy@gmail.com

**Keywords:** doxorubicin-induced cardiotoxicity, glutathione (GSH), oxidative stress, inflammation, cardioprotective mechanisms

## Abstract

Doxorubicin (DOX) is a potent chemotherapeutic agent used to treat many types of cancer. Its use is limited because of the reported accompanied cardiotoxicity, which is driven by oxidative stress and inflammation. Herin, we explored the cardioprotective impact of reduced glutathione (GSH) against DOX-induced cardiac damage in a mice model and highlighted the dynamic interplay between pro-inflammatory and antioxidant mechanisms, with tissue damage markers and oxidative byproducts. Mice were divided into four groups and administered DOX, GSH, or a combination, and the outcomes were compared to untreated controls. DOX administration caused significant mortality, weight loss, elevated serum markers of cardiac injury (CK-MB and LDH), oxidative stress (MDA and iron), pro-inflammatory cytokines (IL-6, IL-17, and IL-23), and upregulated pro-inflammatory gene expression of *STAT-3* and *NFκB* as well as downregulated gene expression of *NRF-2* and HO-1. Histological analysis showed myocardial fibrosis, vacuolization, and apoptosis, as confirmed by a TUNEL assay. Meanwhile, treatment with GSH improved survival rate, attenuated weight loss, and restored cardiac function markers. Furthermore, GSH suppressed oxidative stress and inflammation, modulated gene expression, and declined histopathological damage. These findings demonstrated the multifaceted cardioprotection of GSH through the restoration of redox homeostasis and modulation of the pro- and anti-inflammatory responses. GSH supplementation emerges as a promising adjunct therapy to mitigate DOX-induced cardiotoxicity, offering a strategy to improve cardiac health in cancer patients undergoing doxorubicin chemotherapy.

## 1. Introduction

Doxorubicin (DOX) is a common chemotherapeutic agent for many malignancies, including breast malignancies, lymphomas, and leukemias [1,2,3]. Although it is highly effective in treating rapidly dividing cancer cells, doxorubicin’s therapeutic application is restricted due to well-documented cardiotoxic side effects accompanied with treatment [4,5,6]. This has manifested as irreversible cardiac cells damage resulting in acute or chronic myocardial damage that may ultimately lead to heart failure [7]. DOX-induced cardiotoxicity is a multifactorial process, involving excessive production of reactive oxygen species (ROS) leading to oxidative stress [8], inflammatory signaling [9], and apoptotic pathways [10], as well as mitochondrial dysfunction [11,12] which activates pro-inflammatory pathways that exacerbate tissue damage, which collectively impair cardiac function and viability [13,14]. This is considered as a serious clinical issue, there is a need for elucidating the molecular mechanisms underlying doxorubicin-induced cardiotoxicity. New possibilities for cardioprotection can be achieved through targeting these pathways. Although there are many advances in cardioprotective approaches, effective interventions to alleviate DOX’s harmful effects remain an unmet clinical need [15,16,17,18].

Several compounds have shown promise in preclinical models. Several natural compounds showed promise in protection against doxorubicin-induced cardiotoxicity through mechanisms which diminish oxidative stress, inhibit inflammation, and prevent cell death in cardiac tissue, which enhances cell survival and mitochondrial health [19,20,21,22,23]. These compounds provide a multi-faceted approach to cardioprotection, highlighting their potential as adjunct therapies to reduce doxorubicin’s cardiotoxic side effects.

L-reduced glutathione (GSH) is a water-soluble tripeptide thiol antioxidant. It is the most abundant intracellular small-molecule thiol [24]. It is a crucial antioxidant found in every cell and has a significant role in cellular redox homeostasis through neutralizing ROS and protecting against oxidative stress, detoxifying harmful substances, maintaining cellular health, supporting immune function, and regenerating other antioxidants and modulating inflammatory signaling [25,26]. Preclinical studies suggested that GSH supplementation attenuates oxidative injury in various disease models, yet its potential to counteract DOX-associated cardiotoxicity remains underexplored [27,28]. It exists in a reduced and oxidized form, with the ratio of these forms serving as a significant sign of redox status; a higher GSH level relative to oxidized glutathione (GSSG) reflects better antioxidant capacity [29]. The body synthesizes GSH from amino acids, and it can also be acquired through dietary sources like fruits and vegetables, particularly cruciferous ones [30,31,32,33]. However, as we get older, the first enzyme in the two-step biosynthesis pathway for GSH is compromised. Low levels of GSH have been linked to various health issues, including neurodegenerative diseases and aging, prompting interest in supplementation [34,35].

This study focused on exploring the therapeutic effects of reduced L-glutathione (GSH) against cardiotoxicity induced by doxorubicin, focusing on its role in suppressing inflammatory cascades and modulating redox homeostasis biomarkers and oxidative stress. By elucidating the underlying mechanisms, this research seeks to provide insights into offering novel insights into its therapeutic potential for mitigating cardiovascular side effects and safeguarding cardiac health in cancer patients undergoing anthracycline-based chemotherapy.

## 2. Results

### 2.1. Effect of Dox and GSH on Survival Rate, Body Weight, and Heart Weight for Different Treatment Groups

**Figure 1:**
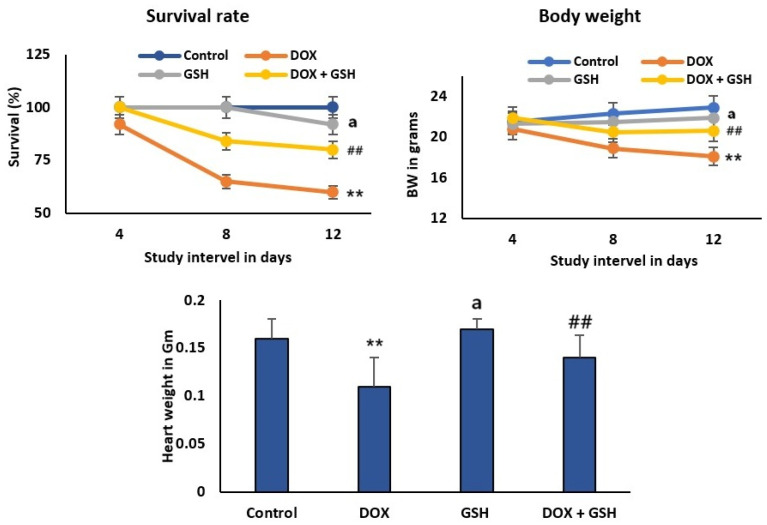
Effect of DOX, GSH, and DOX + GSH treatments on survival rate, body weight, and heart weight in experimental groups. Data represents means ± SEM in each group.** *p* < 0.05 represents significant difference vs. normal control group. ## *p* < 0.05 represents significant difference vs. DOX group. a represents non-significant difference vs. normal control group.

Figure 1 showed the survival percentages of experimental groups over a 12-day study interval. The control and GSH groups maintain high survival throughout the study period, while the survival rate decreased most significantly in the DOX group over time, exhibiting a time-dependent decline and dropping below 75%. Co-treatment with DOX + GSH significantly improved the survival rate compared to DOX alone, maintaining higher survival than the DOX group, suggesting that GSH mitigates DOX-induced toxicity.

Body weight is reduced over time in the DOX group compared to control, indicative of cardiotoxicity. GSH monotherapy resulted in the maintenance of a stable body weight throughout the study with no significant change, whereas DOX + GSH recipients experienced less weight loss compared to treatment with DOX alone and heart weight was restored towards control levels, highlighting GSH’s cardioprotective effect. However, the relative heart weight/body weight ratio was significantly elevated in the DOX group (0.68 ± 0.03%) if compared to the control group (0.61 ± 0.02%). Meanwhile, treatment with GSH attenuated this increase and restored the ratio (0.63 ± 0.02) close to the control group. In addition, the GSH group is comparable to the control group, showing no adverse effects on heart weight.

### 2.2. Effect of Dox and GSH on Serum and Tissue Creatine Kinase-MB (CK-MB) and Serum Lactate Dehydrogenase (LDH)

**Figure 2:**
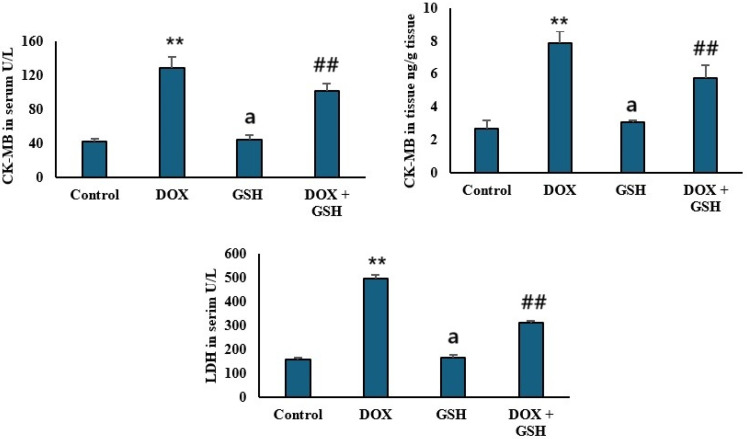
Analysis of cardiac biomarkers in response to different treatments. The upper left panel shows serum levels of CK-MB (U/L) for the control, DOX, GSH, and DOX + GSH groups. The upper right panel presents CK-MB levels (ng/mg tissue) in cardiac tissue for the same groups. The lower panel displays LDH levels (U/L) in serum across all treatment groups. Data are presented as the mean ± SEM. ** *p* < 0.05 represents a significant difference vs. the normal control group. ## *p* < 0.05 represents a significant difference vs. the normal control group. a represents a non-significant difference vs. the DOX group.

DOX administration significantly elevated serum and tissue CK-MB, a marker of myocardial injury, compared to control. GSH co-treatment (DOX + GSH) attenuated this incline and restored serum and tissue CK-MB near control values. The GSH only group shows levels comparable to the control. The GSH group maintains levels similar to the control group, indicating no adverse effects as shown in Figure 2. Furthermore, serum Lactate Dehydrogenase (LDH), as a marker for tissue damage, is markedly elevated in the DOX group. Co-treatment with GSH (DOX + GSH) reduced LDH activity toward control levels. The GSH group maintained LDH levels comparable to the control group.

### 2.3. Malondialdehyde (MDA) Levels and Iron Content in Tissue

**Figure 3:**
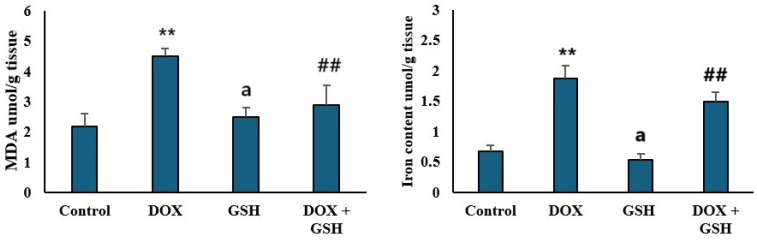
Assessment of oxidative stress markers and iron content in tissue. The left panel displays malondialdehyde (MDA) levels (μmol/g tissue) for the control, DOX, GSH, and DOX + GSH groups. The right panel shows iron content (μmol/g tissue) in the same treatment groups. Data are presented as the mean ± SEM. ** *p* < 0.05 represents a significant difference vs. the normal control group. ## *p* < 0.05 represents a significant difference vs. the normal control group. a represents a non-significant difference vs. the DOX group.

DOX treatment markedly elevated iron content and MDA levels, biomarkers of lipid peroxidation, compared to the control group, indicating oxidative stress. GSH monotherapy (GSH) resulted in MDA levels and iron content comparable to the control, showing no increase in oxidative stress. Co-administration of GSH with DOX (DOX + GSH) significantly reduced MDA and iron content, demonstrating GSH’s ability to counteract DOX-induced lipid peroxidation and oxidative stress as shown in Figure 3.

### 2.4. Pro-Inflammatory Cytokines Levels (IL-6, IL-17, and IL-23)

**Figure 4:**
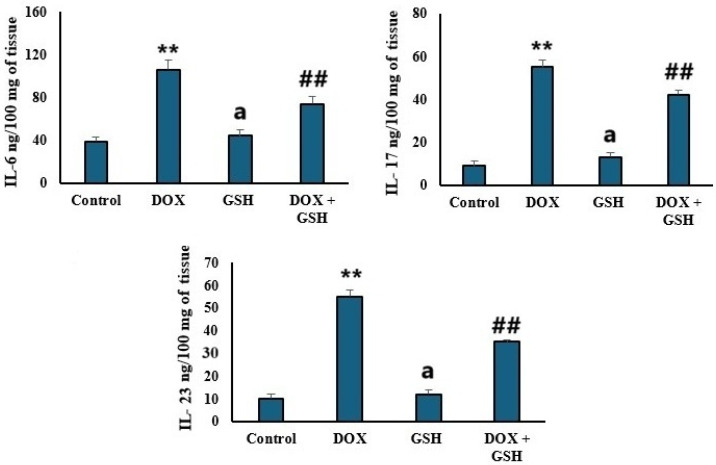
Cytokine levels in tissue following different treatments. The upper left panel shows interleukin-6 (IL-6) concentrations (ng/100 mg of tissue). The upper right panel presents interleukin-17 (IL-17) levels (ng/100 mg of tissue). The lower panel displays interleukin-23 (IL-23) concentrations (ng/100 mg of tissue). Data are presented as the mean ± SEM. ** *p* < 0.05 represents a significant difference vs. the normal control group. ## *p* < 0.05 represents a significant difference vs. the normal control group. a represents a non-significant difference vs. the DOX group.

Figure 4 showed that DOX treatment significantly elevated IL-6, IL-17, and IL-23 levels compared to the control group indicating enhanced inflammatory activity. GSH alone (GSH group) maintains cytokines levels comparable to the control. Co-administration of GSH with DOX (DOX + GSH) significantly reduced cytokines.

### 2.5. mRNA Expression Levels of STAT-3, NRF-2, NFκB, and HO-1

**Figure 5:**
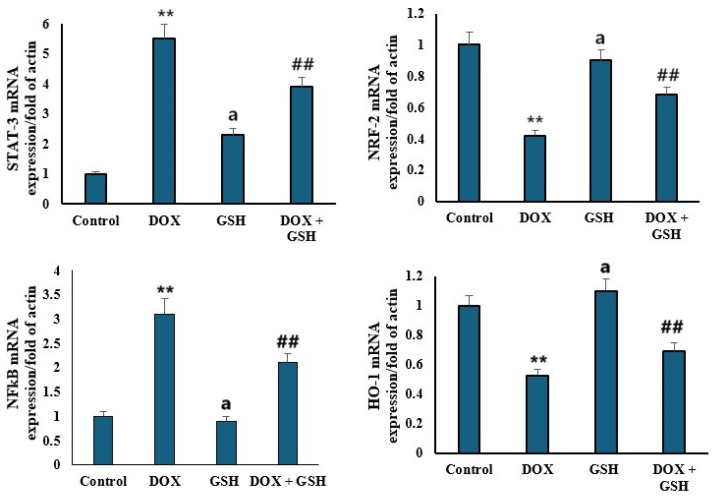
Expression levels of key mRNA markers in tissue following various treatments. The upper left panel shows STAT3 mRNA expression. The upper right panel presents NRF2 mRNA expression. The lower left panel displays NF-κB mRNA expression. The lower right panel shows HO-1 mRNA expression in the DOX, GSH, and DOX + GSH groups, normalized to β-actin. Data are presented as the mean ± SEM. ** *p* < 0.05 represents a significant difference vs. the normal control group. ## *p* < 0.05 represents a significant difference vs. the normal control group. a represents a non-significant difference vs. the DOX group.

Figure 5 illustrated the relative mRNA expression levels of signal Transducer and Activator of Transcription 3 (*STAT-3*), Nuclear factor erythroid 2-related factor 2 (*NRF-2*), Nuclear Factor kappa-light-chain-enhancer of activated B cells (*NFκB*), and heme oxygenase-1 (HO-1) normalized to actin across four experimental groups: control, DOX, GSH, and DOX + GSH. Compared to the control group, DOX treatment markedly increased *STAT-3* and *NFκB* mRNA expression, whereas the GSH group maintains *STAT-3* and *NFκB* levels similar to the control group. Treatment with GSH attenuated the DOX-induced upregulation of *STAT-3* and *NFκB* compared to DOX alone. Furthermore, the DOX group exhibited a reduction in *NRF-2* and HO-1 expression compared to the control. Both GSH and DOX + GSH groups show higher *NRF-2* and HO-1 levels compared to DOX alone, with the GSH group having the highest expression, close to the control group. Moreover, a significant negative correlation between HO-1 expression and iron content with r = −0.72, *p* < 0.05, was noticed. Thus, higher HO-1 expression correlated with lower iron accumulation in cardiac tissue.

### 2.6. Histological Examination

**Figure 6:**
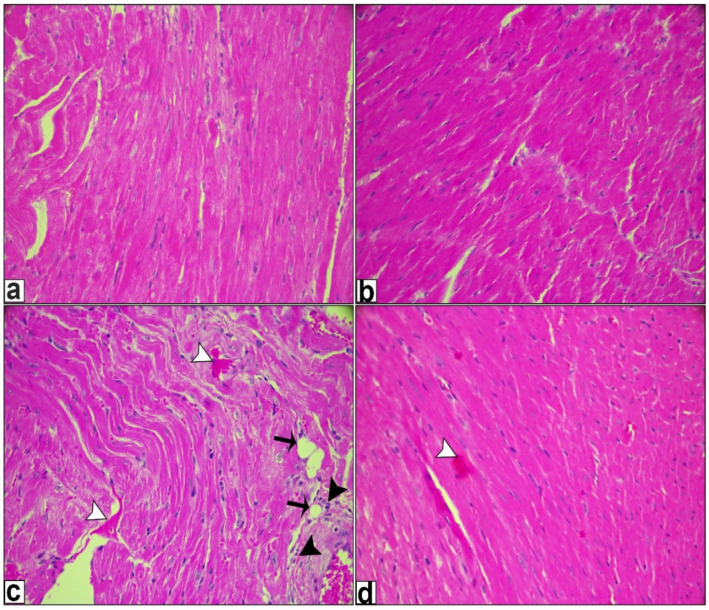
Representative H&E staining of mice heart. (**a**,**b**) refers to the control and reduced-glutathione-treated groups, respectively, that showed a standard architecture in the heart samples. (**c**) The Dox-treated group displayed a small cluster of myocardial fibers with small and large cytoplasmic vacuoles (black arrow), hemorrhage (white arrowheads), and inflammatory infiltration (black arrowheads). (**d**) Dox + reduced-glutathione-treated animals revealed recovery of the myocardial pattern with minor hemorrhage spots. (Magnification ×400).

Hematoxylin and eosin staining revealed an almost standard structure for the heart samples of both the control and reduced-glutathione-treated groups, as seen in Figure 6a,b, while DOX treatment resulted in a clear disorder of the cardiac tissue structure, increased cytoplasmic vacuolization, myofibrillar loss, and an increase in the region of myocardial fibrosis, as demonstrated by H&E in Figure 6c. On the other hand, reduced glutathione treatment successfully decreased the extent of myocardial fibrosis, decreased cytoplasmic vacuolation and myofibrillar loss, and enhanced tissue organization. When combined, these findings indicated that reduced glutathione could be able to reduce the myocardial fibrosis and inflammation brought on by DOX in vivo as shown in Figure 6d. Reduced GSH treatment resulted in a reduction in inflammatory markers and a decrease in the area of fibrosis, alongside an enhancement in cardiac function, when compared to mice treated with DOX.

**Figure 7:**
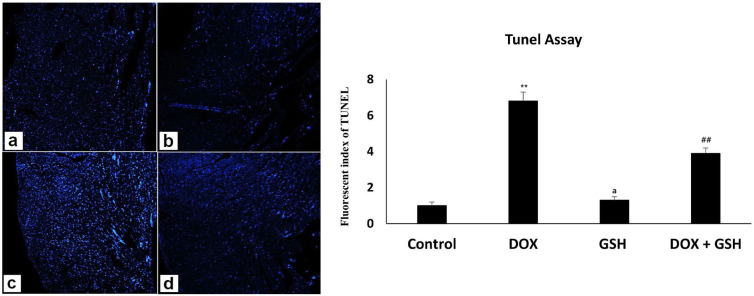
Tunnel assay displayed apoptotic cell death (blue color) with a minor-to-moderate count in the case of both the control and reduced-glutathione-treated groups (**a**,**b**), while DOX-treated samples revealed an intense count of blue apoptotic cell deaths (**c**); in contrast, DOX + reduced-glutathione-treated animals displayed a reduced count of apoptotic cell deaths (**d**). The Tunnel assay analysis chart showed elevated apoptotic cell death within the DOX-treated group when compared with the control and/or GSH-treated group, while this count was decreased in the DOX + GSH-treated group (Magnification ×200). Representative chart for fluorescent staining quantitative analysis expression (n = 5 per group). All data are represented as the mean ± SEM. ** *p* < 0.05 represents a significant difference vs. the normal control group. ## *p* < 0.05 represents a significant difference vs. the normal control group. a represents a non-significant difference vs. the DOX group.

In the TUNEL assay, the control and reduced-glutathione-treated groups showed a minor count of apoptotic cell deaths as displayed in Figure 7a,b; however, DOX treatment significantly raised apoptotic cell death expression, as seen by the elevated blue color in Figure 7c. However, apoptosis was significantly reduced by reduced glutathione co-treatment, as seen in Figure 7d. Accordingly, when comparing the DOX + GSH treated group to the DOX-treated group, the representative chart for fluorescence staining quantitative analysis expression shows a decrease in the number of apoptotic cell deaths, indicating that GSH has a mitigating effect on cell death.

## 3. Discussion

The present study demonstrated the critical role of glutathione (GSH) in mitigating doxorubicin (DOX)-induced toxicity. The survival rate of rats highlighted an absolute contrast between the DOX monotherapy group and the group that received a GSH supplement. The high decline in survival observed in the DOX group aligns with the established literature documenting DOX’s dose- and time-dependent cardiotoxicity, which often limits its clinical utility [36,37]. However, the significant improvement in survival with DOX + GSH co-treatment highlights the protective potential of GSH through its antioxidative properties.

Furthermore, the body weight improvement further supported GSH’s protective capacity. The marked weight loss in the DOX group is consistent with reports of DOX-induced metabolic dysregulation; as explained previously, DOX decreases glucose uptake of adipocytes and serum adiponectin as well as some lipogenic and adipogenic factors in adipose tissue [38,39]. In contrast, the constant body weight in the case of the GSH-only group and the diminished weight loss in the DOX + GSH group imply that GSH maintains metabolic homeostasis and alleviates the tissue damage driven by DOX.

The monitored attenuation of heart weight loss in the DOX + GSH group suggested that GSH may target specific pathways of DOX toxicity. For instance, DOX is well known for promoting iron-mediated free radical production and disrupting calcium homeostasis in heart tissue, which results in apoptosis and fibrosis. However, GSH reduces the effect reactive oxygen species (ROS) and enhances detoxification, thereby preserving cardiac mass and function [40,41]. Notably, the absence of harmful cardiac consequences in the GSH-only group validated its safety, encouraging its suitability as a therapeutic agent [42]. The increased relative heart weight/body weight ratio in the DOX group indicated cardiac hypertrophy and dilated cardiomyopathy (DCM)-like pathology, which is consistent with DOX-induced cardiotoxicity [43,44]. However, GSH treatment significantly attenuated this increase, demonstrating cardioprotection. This suggests that GSH mitigates DOX-induced cardiac hypertrophy, a hallmark of pathological remodeling.

The elevated level of serum and tissue CK-MB and LDH in the DOX group reflected the cardiomyocyte damage and tissue injury [45]. This is consistent with the well-documented cardiotoxic effects of anthracyclines [46,47]. It has been previously documented that Dox disrupts the myocardial cell membrane’s integrity and induces leakage of intracellular enzymes due to oxidative stress, cellular necrosis, and apoptosis in cardiac tissues as well as mitochondrial dysfunction [48,49]. In the present study, co-administration of GSH with DOX restored CK-MB and LDH levels close to the control group. The cardioprotective effects of GSH were likely realized through its antioxidant properties, which neutralized ROS and preserved membrane stability.

In the present study, the apparent increase in malondialdehyde (MDA) and iron content in the DOX group emphasized the key role of iron in driving oxidative stress and lipid peroxidation. DOX is known to interrupt iron homeostasis, which stimulates Fenton reaction-mediated ROS production leading to lipid peroxidation and membrane damage [50,51]. The significant decline in MDA and iron content upon treatment with GSH highlighted the dual capacity of GSH to reduce excess iron and decrease ROS production. The current study displayed excessive Fe^2+^ accumulation in cardiomyocytes in DOX-treated mice which can lead to lipid peroxidation, subsequently triggering ferroptosis (an iron-dependent lipid peroxidation process) that builds up harmful lipid peroxides and reactive oxygen species (ROS), which eventually cause cell death [52]. Ferroptosis is a significant factor in the development of DOX-induced cardiomyopathy [53,54]. On the contrary, the reduced iron content in the cardiac tissue of mice treated with GSH + DOX inhibits lipid peroxidation and preserves iron homeostasis, alleviating DOX’s harmful effects.

Previous studies explored the role of oxidative stress in activating pro-inflammatory pathways [55,56,57]. The elevated pro-inflammatory cytokine profile (IL-6, IL-17, and IL-23) in the DOX group aligns with ROS-mediated activation of inflammatory cascades, which intensifies tissue injury. This was explained previously as DOX accumulates in cardiac mitochondria due to its affinity for cardiolipin, which causes disruption of the electron transport chain and leads to increased ROS generation [9,58]. In the present study, GSH suppressed cytokine elevation in the DOX + GSH group, which confirmed its anti-inflammatory properties, likely mediated through inhibition of redox-sensitive transcription factors such as *NFκB* [59]. *STAT-3* and *NFκB* are key mediators of inflammation and oxidative stress. Upon treatment with DOX, upregulation of *STAT-3* and *NFκB* was observed which reflects their activation in response to cellular damage. However, GSH co-treatment attenuated this upregulation, likely by disrupting the ROS-dependent signaling that drives their transcriptional activity. On the contrary, the downregulation of *NRF-2* and HO-1 in the DOX group demonstrated the impaired antioxidant defense systems. GSH restored *NRF-2* and HO-1 expression, underscoring its role in activating the *NRF-2*/ARE pathway which is a key regulator of cellular antioxidant responses [60,61]. *NRF-2* mediated HO-1 expression and thus enhanced the degradation of pro-oxidant heme to produce cytoprotective molecules like bilirubin [62]. This study revealed a significant negative correlation between HO-1 and iron content. Thus, higher HO-1 expression correlated with lower iron accumulation in cardiac tissue. This suggests that GSH-induced HO-1 upregulation may mitigate iron-mediated oxidative stress by enhancing iron sequestration or export mechanisms.

According to the results of this study, DOX induced ROS production which enhanced redox-sensitive transcription factors (e.g., *NFκB*) leading to the release of pro-inflammatory cytokines (IL-6 and IL-17). On the other hand, IL-6 mediated the inflammation and amplified the oxidative stress by suppressing antioxidant defenses (e.g., *NRF-2*/HO-1). Fortunately, GSH disrupted this interplay by scavenging ROS, inhibiting *NFκB* activation, and upregulating *NRF-2* to restore antioxidant capacity. GSH’s dual modulation highlighted its potential for targeting the inflammation–oxidative stress axis.

Compared to mice treated with DOX, this study showed that reduced GSH inhibited IL-6 which is a proinflammatory cytokine that plays a significant role in doxorubicin-induced cardiotoxicity in the myocardium. This was confirmed by a reduced fibrosis area as well as improved cardiac function. Also, reduced GSH treatment for DOX-treated animals was shown to improve most of the cardiac profiles against DOX-induced cardiotoxicity and this was in congruence with the results of Lee et al. [63].

The present investigation demonstrated a discernible recovery in the TUNEL assay cell count for Dox-induced cardiotoxicity when treated with reduced glutathione, indicating the useful function of reduced glutathione in ameliorating Dox-induced cardiotoxicity. Crucially, the cells labelled with TUNEL were cardiomyocytes, indicating that the primary cause of DOX-induced cardiotoxicity was cardiomyocyte mortality. Doroshow and his team presented similar findings, showing that GSH peroxidase1 modulates intracellular levels of doxorubicin-induced reactive oxygen species (ROS), which are crucial for doxorubicin-induced apoptosis and altered cell cycle progression in murine cardiac fibroblasts [64].

While our findings highlighted the cardioprotective role of GSH, limitations should be mentioned. The short-term study duration (14 days) prevents assessment of the effects of chronic GSH supplementation or delayed cardiotoxicity. Thus, future work should spotlight human cardiomyocyte models or large-animal studies to justify these findings, followed by phased clinical trials to evaluate translational efficacy. Chronic GSH supplementation could suppress endogenous synthesis through feedback inhibition of γ-glutamylcysteine ligase. Thus, future work has to be conducted to assess long-term GSH supplementation and incorporate multi-organ histopathology and serum redox balance markers to outline the GSH therapeutic window.

## 4. Materials and Methods

### 4.1. Animal Study and Biochemical Analysis

A total of 28 C57BL/6 male mice (7–8 weeks old, 23 g) were bred with their pedigree maintained and housed in controlled conditions at 25 °C with a 12 h light–dark cycle and fed a standard diet. All experimental procedures were approved by the Animal Care and Use Committee, Dean ship of scientific research, King Faisal University, Saudi Arabia (Approval number: KFU-REC-2024-DEC-ETHICS2965).

The 28 mice were randomly assigned into 4 groups (n = 7/group): Group-1(Ctrl): healthy mice received saline; Group-2 (DOX): DOX-treated group; Group-3 (GSH): reduced-glutathione-treated group; and Group-4 (DOX + GSH): DOX and reduced-glutathione-treated group.

DOX-induced cardiotoxicity was generated by administering 4 doses (2 mg/kg dissolved in sterile 5% DMSO) on alternative days during the first week. The human oral dose of GSH for an adult of 60 Kg is 500–2000 mg/day; after performing dose conversion to a mouse, the equivalent dose is 102.78–411.11 mg/kg/day when using the body surface area normalization method [65]. Mice divided into the GSH or DOX + GSH group were treated with reduced glutathione (200 mg/kg/day) by oral gavage for alternative days for 14 days. At the end of the experiment, mice were fasted for 12 h following the last dose and sacrificed for histological, biochemical, and molecular marker analysis [66].

### 4.2. CK-MB and LDH Assay

The cardiac marker enzymes Creatine Kinase-MB (CK-MB) (Cat. No.: E-EL-R1327 Product Name: mammalian CKMB) [67] and Lactate Dehydrogenase (LDH) (EEA013, Invitrogen, Waltham, MA, USA) were used [68]. Serum levels and tissue homogenate were assessed using commercial kits according to the manufacturer’s protocols.

### 4.3. Iron Content in Tissue

Protein content in tissue lysates was calculated using the Bradford assay using the manufacturer’s instructions [69]. For iron detection, mixtures of samples and standards with working solution were centrifuged at 20,000 rpm for 5 min. Then, 200 μL were removed into a 96-well plate to record absorbance at 595 nm using a SpectraMax 190 microplate reader (Molecular Devices, San Jose, CA, USA). Iron concentrations were interpolated from the standard curve generated from the iron standards and normalized to the protein content (nmole of iron/mg protein) [70].

### 4.4. Cytokine Estimation

Blood samples were obtained from DOX-induced mice, and the serum was separated using centrifugation. ELISA kits (Invitrogen, USA) were utilized following the manufacturer’s instructions to assess serum interleukin-6 (IL-6), interleukin-17 (IL-17), and interleukin-23 (IL-23) cytokines [71,72].

### 4.5. Quantitative Real-Time PCR

Total RNA was isolated from heart tissues using TRIzol^®^ reagent and reverse transcribed with a First Strand cDNA Synthesis Kit (Thermo Scientific, Invitrogen, Waltham, MA USA) [73]. RT-qPCR was performed with a real-time PCR detection system (Applied Biosystems, Foster City, CA, USA), using the Light Cycler 480 SYBR Green Master Mix. The primers used are listed in Table 1.

### 4.6. Microscopic Examination (H and E Staining) and TUNEL Assay

PBS (phosphate-buffered saline) was used to clean heart samples. After fixing the samples in 4% paraformaldehyde, the tissue was embedded in paraffin and divided into sections that were 3 μm thick [74]. The slices were imaged using a phase contrast microscope (Nikon, Tokyo, Japan) after being stained with hematoxylin and eosin (H&E), and for a one-step TUNEL, staining was performed to detect in situ DNA fragmentation using the in-situ Apoptosis Detection Kit (E-CK-A331, Elabscience, Wuhan, China), using a fluorescence microscope (Olympus DX51, Tokyo, Japan) [75]. The nucleus of TUNEL-positive cells displayed blue stained granules. The number of TUNEL-positive nuclei and the percentage of cardiac fibrosis were detected using Image J software 1.54v (NIH Image, Bethesda, MD, USA).

### 4.7. Statistical Analysis

The mean ± SEM is used to express all data. GraphPad Prism 3.0 (GraphPad Software, Boston, Massachusetts USA) and SPSS software 17.0 (Chicago, SPSS Inc., USA) were used to assess the differences between experimental groups using unpaired or paired *t*-tests. A significant level of *p* < 0.05 was established. A post hoc power analysis confirmed >85% power to detect significant differences (α = 0.05) for primary outcomes, based on observed effect sizes (Cohen’s d > 1.8).

## 5. Conclusions

This study displayed that GSH mitigated DOX-induced cardiotoxicity. Upon treatment with GSH, survival rates, body and heart weight loss, CK-MB and LDH levels were restored. GSH suppressed lipid peroxidation through reducing MDA and decreased ferroptosis by declining iron content in cardiac cells. Furthermore, GSH decreased IL-6, IL-17, and IL-23 cytokines which highlighted its anti-inflammatory activity. It inhibited *STAT-3* and *NFκB* and activated *NRF-2* and HO-1, which stimulated cellular defense mechanisms. The histopathological study showed reduced myocardial fibrosis and vacuolization in the GSH-treated groups. The current study establishes GSH supplementation as a potential co-therapeutic approach to enhance the safety of chemotherapy protocols involving DOX.

## Figures and Tables

**Table 1:** List of primer sequences.

Gene Name	Forward Sequence	Reverse Sequence
*STAT-3*	AGGAGTCTAACAACGGCAGCCT	GTGGTACACCTCAGTCTCGAAG
*NRF-2*	GGCAACAGTAGCCACATTGGCT	GTCTGGATGGTCATTTCACCGC
NFκB	GCTGCCAAAGAAGGACACGACA	GGCAGGCTATTGCTCATCACAG
HO-1	CCAGGCAGAGAATGCTGAGTTC	AAGACTGGGCTCTCCTTGTTGC
GAPDH	CATCACTGCCACCCAGAAGACTG	ATGCCAGTGAGCTTCCCGTTCAG

## Data Availability

Data are available from the corresponding author, upon request.
